# Expression and Prognosis Value of the KLF Family Members in Colorectal Cancer

**DOI:** 10.1155/2022/6571272

**Published:** 2022-03-19

**Authors:** Zhongting Huang, Haibin He, Feng Qiu, Hailong Qian

**Affiliations:** Department of Gastrointestinal Surgery, Ningbo Medical Center Lihuili Hospital, Ningbo, Zhejiang, China

## Abstract

Krüppel-like factors (KLFs) are some kind of transcriptional regulator that regulates a broad range of cellular functions and has been linked to the development of certain malignancies. KLF expression patterns and prognostic values in colorectal cancer (CRC) have, however, been investigated rarely. To investigate the differential expression, predictive value, and gene mutations of KLFs in CRC patients, we used various online analytic tools, including ONCOMINE, TCGA, cBioPortal, and the TIMER database. KLF2-6, KLF8-10, KLF12-15, and KLF17 mRNA expression levels were dramatically downregulated in CRC tissues, but KLF1, KLF7, and KLF16 mRNA expression levels were significantly elevated in CRC tissues. According to the findings of Cox regression analysis, upregulation of KLF3, KLF5, and KLF6 and downregulation of KLF15 were linked with a better prognosis in CRC. For functional enrichment, our findings revealed that KLF members are involved in a variety of cancer-related biological processes. In colon cancer and rectal cancer, KLFs were also shown to be associated with a variety of immune cells. The findings of this research reveal that KLF family members' mRNA expression levels are possible prognostic indicators for patients with CRC.

## 1. Introduction 

Colorectal cancer (CRC) is the third most common cancer in the world and the second main cause of cancer-related deaths. There were approximately 1.9 million new cases of CRC worldwide, and over 910,000 of these patients died of CRC in 2020 [1]. Despite significant efforts in early detection and treatment options, the prognosis for people with CRC remains poor. About 50% of patients with CRC present with metastasis or recurrence, which hinders the therapeutic efficacy of CRC treatment [2]. As a result, it is critical to find useful biomarkers that can predict CRC patients' clinical outcomes and act as therapeutic targets for CRC therapy.

Krüppel-like factors (KLFs) are a transcription factor family that includes 17 members that feature zinc finger domains. Three cys2/his2 zinc finger DNA binding domains that regulate gene transcription via binding to GC-rich DNA sequences and 5-CACCC-3 elements distinguish these evolutionarily conserved KLF members. KLFs are required for the proper functioning of various biological cellular processes, including proliferation, differentiation, apoptosis, and migration [3]. During the past few years, numerous evidence indicated that the expression of several KLF family members was dysregulated in many human malignancies, where they can serve as tumor suppressors and/or oncogenes in cancer-specific cellular contexts [4]. Recently, increasing research studies have also identified KLFs as novel prognosis markers in human cancers. For example, KLF4 was demonstrated as a tumor suppressor in lung cancer, Hu et al. reported that the expression of KLF4 was decreased in most lung cancer tissues, and upregulation of KLF4 resulted in marked inhibition of cell growth and colony formation [5]. In contrast, the oncogenic activity of KLF4 was discovered in breast cancer, where higher KLF4 expression was seen in primary breast ductal carcinoma compared to normal tissues [6]; moreover, overexpression of KLF4 led to a poor prognosis in early-stage breast cancer. Overexpression of KLF6 in prostate cancer inhibits proliferation and induces apoptosis, showing that KLF6 functions as a prostate tumor suppressor [7, 8]. KLF7 overexpression was related to a worse prognosis in gastric and lung cancers, according to studies [9, 10]. Additionally, Liu et al. reported that KLF17 inhibited hepatocellular carcinoma (HCC) cell motility and invasion possibly via counteracting EMT, and reduced expression of KLF17 was associated with poor prognosis in HCC [11]. KLF8 has been shown to promote CRC invasion and metastasis by activating FHL2 through transcriptional regulation [12]. KLF14 can regulate LDHB through transcriptional regulation and thus affect glycolysis [13].

Previous research has shown the expression patterns of individual KLFs in CRC and their link to CRC patients. Still, the overall picture of the KLF family in CRC is yet to be fully analyzed, which is urgently needed. The present work analyzed the expression status and prognostic values of the whole KLF family in CRC using updated public databases, allowing for a better knowledge of this huge family and the development of novel therapeutic drugs.

## 2. Materials and Methods

### 2.1. Analysis of the ONCOMINE Database

The ONCOMINE database (https://www.oncomine.org), an open public database that enables sophisticated genome-wide expression analysis, was used to determine the expression levels of KLF family genes. The following levels of significance were used in this study: *P* < 0.05, |logFC| ≥ 1, and expressed genes ranking in the top 10%. The *p* value for the difference in KLF gene expression in CRC was calculated using Student's t-test.

### 2.2. Analysis of the TCGA Database

In our study, 647 CRC tissues and 51 normal controls were obtained from the Cancer Genome Atlas (TCGA) database. Subsequently, matched clinical parameters including gender, age, stage, T classification, N classification, M classification, survival status, and overall survival (OS) time were extracted by using the GDC Data Transfer Tool for secondary analysis. Using the corrplot program in R 3.6.0 software, we analyzed the correlation between KLF family genes using Pearson's method. The limma package was used to look at the differences in KLF family mRNA expression between CRC tissues and normal controls (FPKM value). The pheatmap and ggpubr packages were used to visualize the findings.

### 2.3. KLF Gene Genetic Alteration Analysis

cBioPortal is a web-based tool for exploring, visualizing, and interpreting multimodal cancer genomic data. cBioPortal for Cancer Genomics was used to investigate the KLF genes and gene mutations in CRC.

### 2.4. MRNA Expression and Methylation of KLF Members in CRC

The Illumina Human Methylation 450K Array of TCGA-CRC project was downloaded from UCSC Xena (https://xenabrowser.net). The *β* values of CG sites in the promoter region of KLF genes in CRC were extracted using Perl 5.26 software. Afterwards, the correlations between DNA methylation levels and mRNA expression levels among KLF family genes in CRC tissues were analyzed by the corrplot package.

### 2.5. Analysis of Prognostic Values of KLF Genes in CRC

According to the optimal cutoff point, CRC patients with integrated survival data were separated into high and low groups. The Kaplan–Meier approach was used to construct OS curves, and log-rank tests were used to determine the difference between the survival curves. Following that, a univariate Cox proportional hazard regression analysis was conducted to identify KLF genes and to filter out genes that were substantially linked with OS. By univariate and multivariate regression analysis, we obtained genes with independent predictive value for the KLF gene family.

### 2.6. Analysis of TIMER Database

We used the TIMER database's gene module to validate the link between KLF gene expression and the amount of immune cell infiltration in colon and rectal cancer [14].

### 2.7. GSEA

We used gene set enrichment analysis (GSEA) to deduce the probable processes by which KLF genes contribute to the carcinogenesis of colorectal cancer [15]. TCGA samples were classified into high and low KLF gene expression categories. *P* < 0.05 and a false discovery rate (FDR) of 0.25 were regarded as highly enriched.

## 3. Results

### 3.1. Aberrant mRNA Expression of KLF Family Genes in CRC

**Figure 1:**
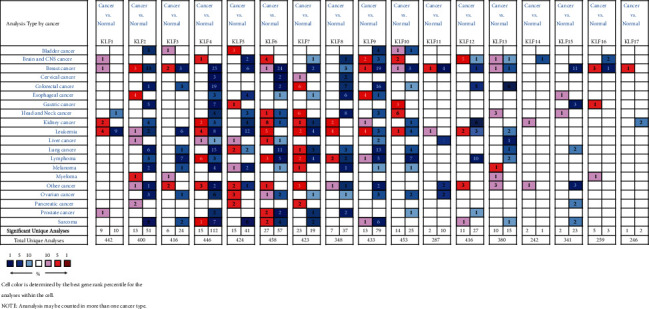
Transcriptional expression of KLF genes in CRC (ONCOMINE database).

**Figure 2:**
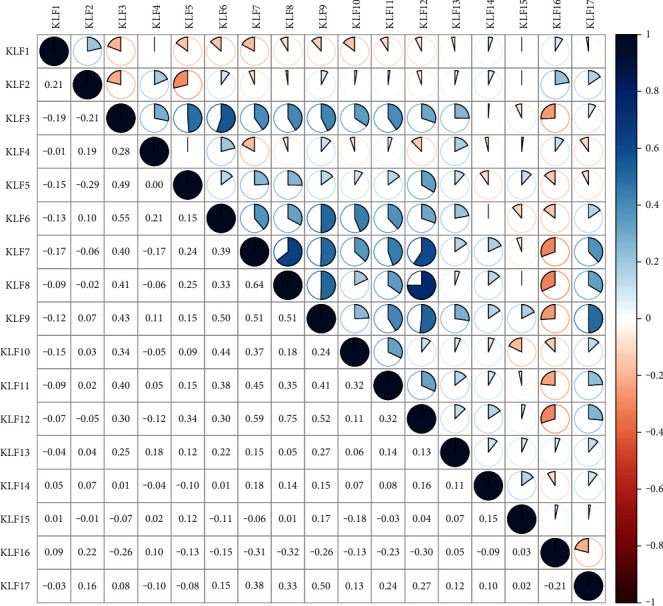
Associations between KLF family genes in CRC.

**Figure 3:**
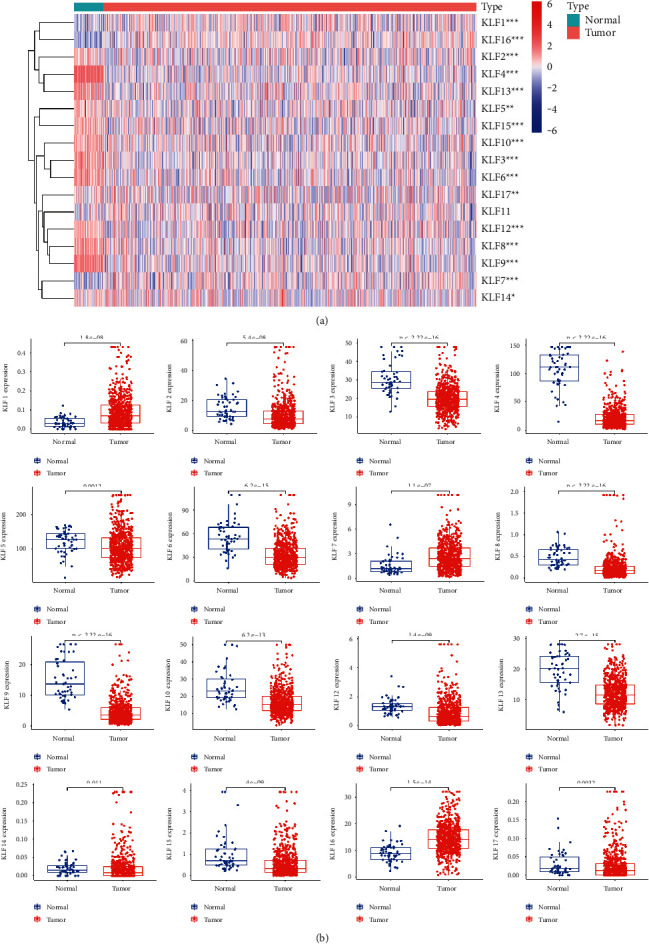
Expression profile of KLF members in CRC. (a) Heatmap of TCGA samples. (b) Histogram of TCGA sample.

The ONCOMINE database was used to look at the transcriptional levels of KLF genes in CRC and healthy control samples. Fold changes (Log2) of mRNA expression of mostly KLF genes were considerably reduced in patients with CRC, as illustrated in Figure 1 and Table S1. Pearson's correlation coefficients of the KLF family genes were then computed to see whether they were connected with each other. The KLF family genes were associated to some extent, as shown in Figure 2. We also analyzed the mRNA expression of KLF family members in CRC and normal tissues using data from the TCGA database. The limma software was used to examine the differentially expressed KLF family genes, and their expression patterns were shown using a heatmap (Figure 3(a)). The expression of KLF11 in CRC and control tissues did not vary significantly.

### 3.2. Differences in KLF Family Gene Variants in CRC Patients

**Figure 4:**
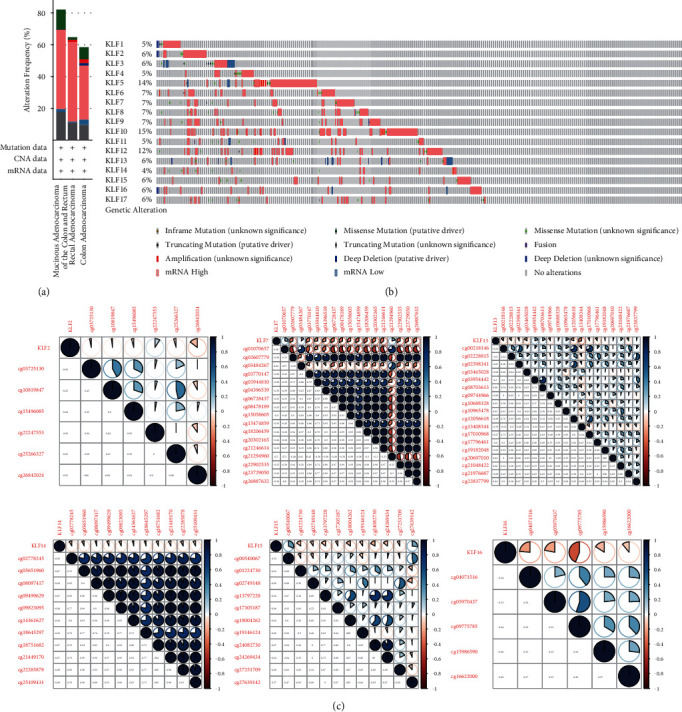
Genetic alterations and association between DNA methylation and expression of KLF genes in CRC. (a) Summary of alterations in differently expressed KLFs in CRC. (b) OncoPrint visual summary of alterations with a query of KLF family members. (c) Association between DNA methylation and expression of KLF genes in CRC.

Genetic modification of 17 genes was done using the cBioPortal database to investigate the involvement of KLF family genes in CRC patients. The results of this study were published in the journal Cancer Research. As seen in Figure 4(b), KLF family genes were found to be altered in 328 samples from 524 CRC patients (63%). KLF10 had the largest genetic variant alteration rate (15%), whereas KLF14 had the lowest (4%). The genetic alteration rates of other KLF family genes signature in CRC were (KLF1, 5%; KLF2, 6%; KLF3, 6%; KLF4, 5%; KLF5, 14%; KLF6, 7%; KLF7, 7%; KLF8, 7%; KLF9, 7%; KLF11, 5%; KLF12, 12%; KLF13, 6%; KLF15, 6%; KLF16, 6%; KLF17, 6%).

### 3.3. Correlation Analysis of KLF Family Gene Promoter Methylation and Expression Level

Using TCGA database samples, we studied the relationship between KLF family gene promoter methylation and expression level in CRC. Almost all CG sites in the promoter region of KLF2, KLF7, KLF13, KLF14, KLF15, and KLF16 had a negative connection with expression in CRC, according to Pearson's correlation results (Figure 4(c)). However, the majority of the CG sites of the other KLF family genes (KLF1, KLF3, KLF4, KLF5, KLF6, KLF8, KLF9, KLF10, KLF12, and KLF17) did not show a negative connection with expression, suggesting that improper DNA methylation may not be the source of the aforementioned genes' aberrant expression.

### 3.4. Overall Survival of KLF Family Gene Signature in CRC

**Figure 5:**
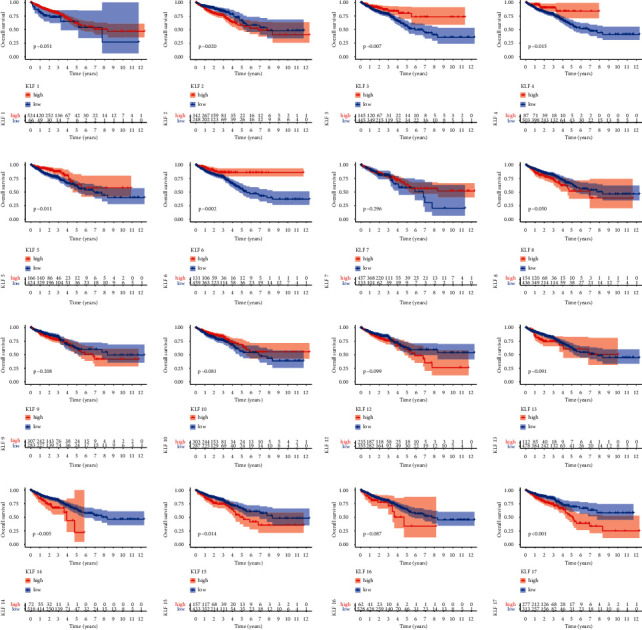
Kaplan–Meier curves of overall survival in patients with CRC with high and low KLF family mRNA expression.

**Figure 6:**
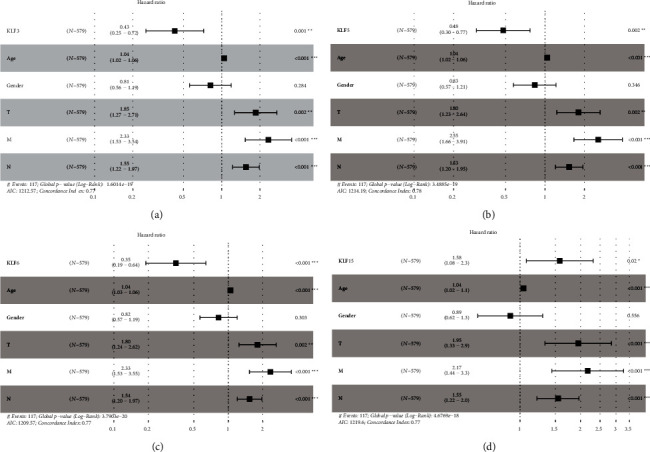
Multivariate Cox proportional hazard regression analysis of KLF members (KLF3 (a), KLF5 (b), KLF6 (c), and KLF15 (d)) and clinicopathologic features for overall survival in CRC.

High mRNA expression of KLF2, KLF14, KLF15, and KLF17 was linked with poor OS, while high expression of KLF3, KLF4, KLF5, and KLF6 was associated with a higher overall survival rate (Figure 5). Following that, the prognostic values of KLF family genes for CRC patients were investigated using the Cox regression method. Univariate analysis showed that KLF2, KLF14, KLF15, and KLF17 were significantly associated with poor OS in patients with CRC, while KLF3, KLF4, KLF5, and KLF6 were significantly associated with better OS in patients with CRC (Table 1). Adjusting for clinical factors (age, gender, T, N, and M stage), multivariate analysis (Figure 6) confirmed that high expressions of KLF3, KLF5, and KLF6 were independent risk factors for better OS in patients with CRC, while high expression of KLF15 was independent risk factor for poor OS in patients with CRC.

### 3.5. Correlation between TIICs and Prognosis-Related KLF Family Genes in Patients with CRC

**Figure 7:**
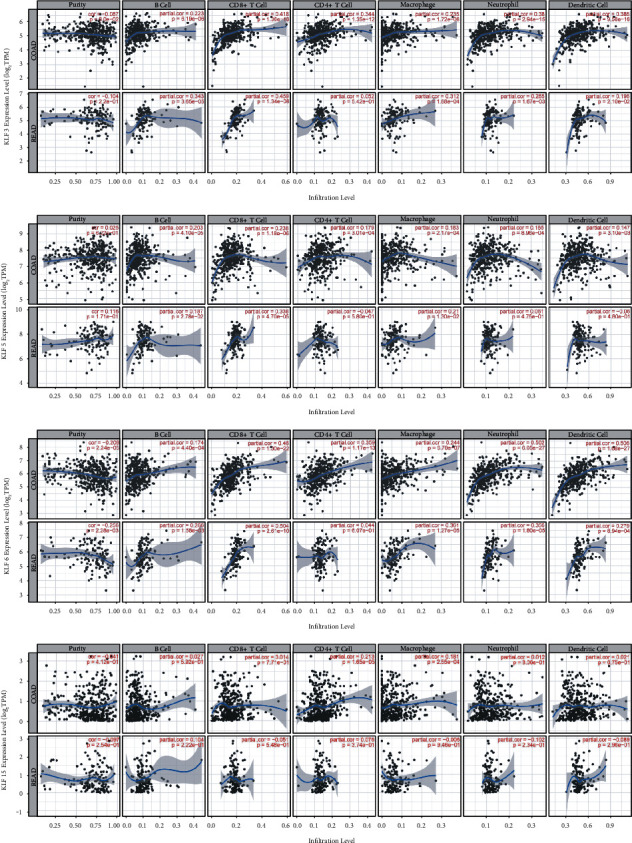
The correlation between KLF members (KLF3, KLF5, KLF6, and KLF15) and the abundance of each type of immune cells (B cells, CD4+ T cells, CD8+ T cells, neutrophils, macrophages, and dendritic cells) using TIMER database.

Because immunological characteristics are linked to cancer prognosis, we used the TIMER database to investigate the relationship between tumor-infiltrating immune cells (TIICs) and prognosis-related KLF family genes in colon and rectal cancer.  Figure 7 depicts the results. The infiltration levels of all TIICs, including B cells, CD4+T cells, CD8+ T cells, neutrophils, macrophages, and dendritic cells, were favourably linked with the expression of KLF3, KLF5, and KLF6 in colon cancer. KLF3 and KLF6 expressions were favourably related to the infiltration levels of all immune cell types except CD4+ T cells in rectal cancer. KLF5 expression was positively related to B cells, CD8+ T cells, and macrophages in rectal cancer. In addition, KLF15 was shown to be positively linked with CD4+ T cell and macrophage levels in colon cancer patients but not in rectal cancer patients.

### 3.6. GSEA

**Figure 8:**
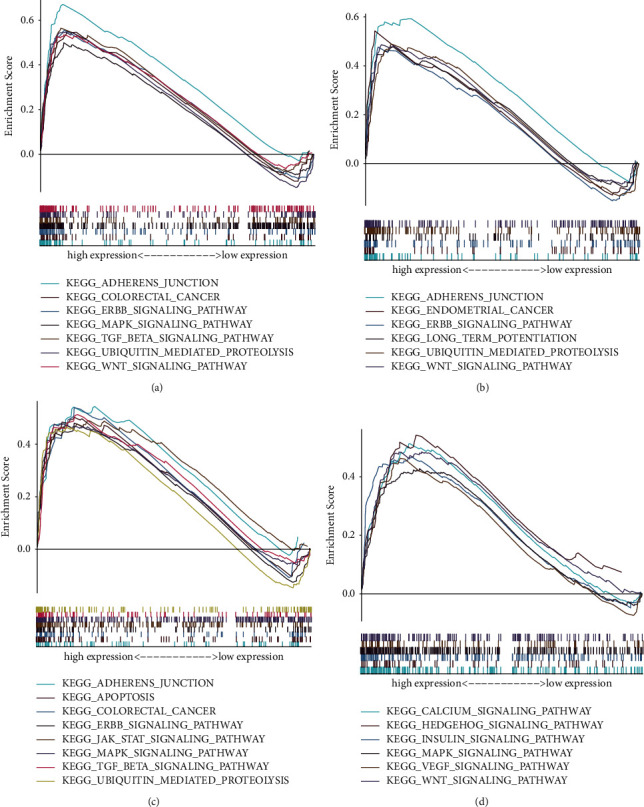
Gene set enrichment analysis (GSEA) of KLF3 (a), KLF5 (b), KLF6 (c), and KLF15 (d) in patients with CRC using TCGA dataset.

To further explore the potential mechanism of KLF family genes affecting the prognosis of CRC patients, we conducted GSEA between increased and decreased KLF3, KLF5, KLF6, and KLF15 expression datasets, which were significant in survival analysis. The results of GSEA are shown in Figure 8.

### 3.7. KLFs and Immune Checkpoints

**Figure 9:**
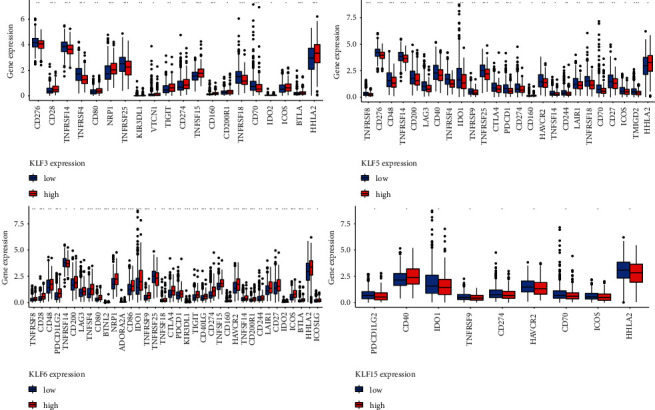
Differences in immune checkpoints between different groups of high and low expressing KLFs.

We grouped the expression of KLF3, KLF5, KLF6, and KLF15 according to their high and low expression and compared the differences in immune checkpoints between the different groups. We found that most of the immune checkpoints differed significantly between the two groups in the high and low expression groups of KLF6 (Figure 9).

## 4. Discussion

KLFs were initially discovered in 1993 as human homologs of the *Drosophila melanogaster* Krüppel protein and given the moniker “Krüppel-like.” The mammalian KLF family now has 17 members, each of which has a conserved C-terminal domain and a substantially divergent N-terminal portion. KLFs are increasingly being proven to play a key role in the promotion and inhibition of a variety of malignancies [16, 17]. Until recently, no publication has provided an overview of how KLF family genes are linked to colorectal cancer. To better understand the clinical practice values of all KLF genes in CRC, we used bioinformatics analysis to examine the expression patterns, prognostic values, and probable function of all KLF family members in CRC.

Previous research has shown that members of the KLF family are overexpressed in tumor tissues or cell lines. KLF5 overexpression has been discovered in gastric cancer, and it has been linked to greater tumor sizes and later tumor (T) stages [18]. KLF8 was shown to be overexpressed in human HCC cell lines and samples from individuals with the disease [19]. KLF15 was shown to be downregulated in gastric cancer but increased in lung cancer [20, 21]. KLF16 expression was much greater in breast cancer tissues than in normal tissues, and knocking down KLF16 greatly reduced cell proliferation in vitro and in vivo. Several investigations have consistently shown that KLF3, KLF4, and KLF6 were significantly downregulated in CRC [22].

In the survival analysis, it was found that increased expression of KLF2, KLF14, KLF15, and KLF17 and decreased expression of KLF3, KLF4, KLF5, and KLF6 in CRC were related to worse OS. KLF3, KLF5, KLF6, and KLF15 were shown to be independent prognostic variables impacting OS in a multivariate Cox proportional hazards regression. A great number of research studies have shown that abnormal KLF expression may also be used to predict patient outcomes. KLF4 has been shown to be a tumor suppressor in a variety of malignancies, and reduced KLF4 expression has been linked to a worse overall survival rate in hepatocellular carcinoma, pancreatic cancer, and renal cell carcinoma [23]. In lung cancer and hepatocellular carcinoma, overexpression of KLF8 was associated with decreased patient survival [24]. KLF17 expression was shown to be lowly expressed in CRC, which was linked to lymph node metastases and poor overall survival, according to recent research [25].

KLF3 regulates tumor cell proliferation, apoptosis, and metastasis and involves modifying the protein that has been discovered in many malignancies. KLF3 expression was shown to be lower in lung tissues, and it was found to be inversely associated with TNM stage and lymph node metastasis. According to in vitro and in vivo research, KLF3 inhibition accelerates lung cancer EMT and promotes lung cancer metastasis through the STAT3 signalling pathway [26]. Overexpression of KLF3 suppressed cell proliferation and increased apoptosis in vitro, and it has also been demonstrated to be downregulated in human pancreatic cancer [27]. In our study, KLF3 expression levels in human CRC were shown to be lower than in normal tissues, and the results of univariate and multivariate Cox regression analysis suggested that KLF3 was associated with a better prognosis in CRC patients. A previous study found that reduction of KLF3 expression is linked to aggressive phenotypes and poor survival outcomes in CRC patients, which is consistent with our results. These findings might point to KLF3 acting as a tumor suppressor gene in CRC.

KLF5's role in cancer is context-dependent; in most malignancies, it serves as an oncogene, whereas in others, it has tumor-suppressive properties [28]. Breast cancer, cervical cancer, hepatocellular carcinoma, and bladder cancer are all aided by KLF5 [29–31]. Furthermore, KLF5 expression has been linked to a poor prognosis in lung cancer, and in vitro tests have shown that a decrease of KLF5 may overcome cisplatin resistance in lung cancer [32]. KLF5 expression was considerably downregulated in esophageal squamous-cell carcinoma compared to normal esophageal epithelial cells, and restoring KLF5 expression activated the JNK pathway, leading to apoptosis and decreased cancer cell survival [33]. Our findings revealed that low KLF5 expression in CRC was linked to a poor prognosis.

KLF6 is extensively expressed in normal tissues, but it is inactivated or downregulated in a variety of human malignancies, most often due to loss of heterozygosity, somatic mutation, promoter hypermethylation, or non-coding RNA suppression [34, 35]. While wild-type KLF6 reduces tumor growth and progression in general, several tumor-derived KLF6 mutations and alternatively spliced variants boost proliferation and tumorigenicity. KLF6 expression in tumors was lower in ovarian cancer than in normal ovarian epithelial cells, while its splice variant KLF6-SV1 expression was higher and correlated with advanced tumor grade [36]. Silencing KLF6 boosted proliferation and invasion, whereas downregulating KLF6-SV1 had the opposite effect. Targeted decrease of KLF6-SV1 expression triggered lung cancer cell death both alone and in conjunction with cisplatin treatment [37]. Overexpression of KLF6-SV1 was related to poor survival in lung cancer. Low KLF6 expression was linked to a poorer prognosis for OS time in patients with CRC in our study, showing that KLF6 works as a tumor suppressor, which is consistent with earlier findings. We found that many immune checkpoints differed between the two groups when patients were divided into high and low expression groups based on KLF6 expression, which also gave us a hint that possibly KLF6 expression may influence immunotherapy.

The role of KLF15 in carcinogenesis is just beginning to emerge. KLF15 has been shown to decrease proliferation in a variety of cancer cells, including pancreatic cancer, endometrial cancer, and breast cancer [38–40]. In lung cancer, Gao et al. found that KLF15 expression was unusually high in cancer tissues and cells when compared to surrounding non-tumorous tissues, and in vitro investigations revealed that lowering KLF15 expression inhibits lung cancer cell proliferation and migration [21]. Qu et al. discovered the involvement of KLF15 in glioma tumor drug resistance, and their functional tests revealed that knocking down KLF15 increased glioma sensitivity to temozolomide cytotoxicity via modulating O6-methylguanine-DNA methyltransferase expression. Our findings revealed that KLF15 was expressed higher in normal tissues than in CRC tissues and that low KLF15 expression was linked to poor CRC patient outcomes. The expression of KLF15 was adversely linked with the methylation level of Cg sites in CRC. As a result, it is easy to deduce that the loss of KLF15 expression is due in part to hypermethylation of its promoter region. Given the paucity of relevant research on the subject, further well-designed studies focusing on the biology and prognostic relevance of KLF15 in CRC are warranted.

It was shown that four KLF genes were strongly related to CRC patients' prognosis. Thus, we used GSEA to investigate the underlying mechanisms of these genes. As a result of the study, it was shown that these genes were involved in the WNT signalling pathway, endometrial cancer, adherens junction, etc., all of which were found to be strongly linked with the incidence and progression of CRC. These findings showed that differentially expressed KLF3, KLF5, KLF6, and KLF15 in CRC might be exploited as potential prognostic indicators and therapeutic targets, either alone or in combination.

Tumor-infiltrating immune cells play a vital role in tumor formation and progression, according to a rising number of research on the tumor microenvironment (TME). There is evidence that TME is high in several kinds of immune cells, which are linked to clinical outcomes and immunotherapy response [41–43]. The expression of KLF3, KLF5, KLF6, and KLF15 in CRC was shown to be highly connected with immune infiltration levels, which was one of the study's key results. Our findings revealed that the transcription levels of these four genes were positively correlated with the levels of infiltration of B cells, CD4+ T cells, CD8+ T cells, neutrophils, macrophages, and dendritic cells. These discoveries might help in the development of novel immunotherapy medications by providing extensive immunological information.

Despite the fact that our research yielded strong findings, there were some limitations. Because the data in our research were RNA expression data acquired from Internet sources, we needed to corroborate our results by looking at changes in protein levels and their prognostic implications. More in vivo and in vitro research studies, as well as clinical studies, are required to confirm our results and investigate the probable processes between KLFs and CRC.

## 5. Conclusion

The expression profiles and prognostic values of KLF family members in CRC were extensively investigated in this in silico work, giving unique insights for future analysis of KLF members as prospective targets in CRC.

## Figures and Tables

**Table 1:** Univariate Cox proportional hazard regression analysis of KLF members and clinicopathologic features for overall survival in CRC.

Variables	Univariate analysis		
	HR	95% CI	*p*
Age	1.036	1.018–1.053	<0.001
Gender	1.086	0.754–1.566	0.658
T	2.649	1.847–3.799	<0.001
N	1.977	1.596–2.450	<0.001
M	3.237	2.234–4.688	<0.001
KLF2	1.558	1.065–2.279	0.022
KLF3	0.487	0.291–0.814	0.006
KLF4	0.408	0.190–0.876	0.021
KLF5	0.580	0.368–0.915	0.019
KLF6	0.384	0.211–0.699	0.002
KLF14	1.838	1.119–3.021	0.016
KLF15	1.593	1.089–2.329	0.016
KLF17	1.858	1.283–2.691	0.001

## Data Availability

The data that support the findings of this study are openly available in the Cancer Genome Atlas (TCGA) program at https://portal.gdc.cancer.gov/.

## Supplementary Materials

Supplementary Materials Table S1: significant changes in KLF expression in transcription level between CRC and normal samples (ONCOMINE database).
