# Supplementary material for: Expression and Prognosis Value of the KLF Family Members in Colorectal Cancer
Source: J Oncol. 2022 Mar 19;2022:6571272. doi: 10.1155/2022/6571272 (PMC8957442; doi:10.1155/2022/6571272)
Supplement: Supplementary Materials — Table S1: significant changes in KLF expression in transcription level between CRC and normal samples (ONCOMINE database). [file 6571272.f1.docx]

Table S1. Significant changes in KLFs expression in transcription level between CRC and normal samples (ONCOMINE Database).

| Gene | Types of CRC vs. Normal Samples | Fold change | P value | t-Test | Dataset |
| --- | --- | --- | --- | --- | --- |
| KLF2 | Rectosigmoid Adenocarcinoma vs. Normal | -11.179 | 2.22E-11 | -12.582 | TCGA Colorectal |
| KLF3 | Rectal Adenocarcinoma vs. Normal | -2.495 | 3.98E-30 | -14.946 | Gaedcke Colorectal |
|  | Rectal Mucinous Adenocarcinoma vs. Normal | -2.133 | 4.44E-07 | -9.851 | TCGA Colorectal |
|  | Colon Adenocarcinoma vs. Normal | -2.028 | 2.64E-16 | -13.338 | TCGA Colorectal |
| KLF4 | Colorectal Carcinoma vs. Normal | -6.405 | 1.34E-29 | -18.799 | Hong Colorectal |
|  | Colon Adenocarcinoma vs. Normal | -2.774 | 7.18E-16 | -12.129 | Kaiser Colon |
|  | Cecum Adenocarcinoma vs. Normal | -2.92 | 3.74E-10 | -11.858 | Kaiser Colon |
|  | Rectosigmoid Adenocarcinoma vs. Normal | -4.94 | 1.34E-06 | -7.894 | Kaiser Colon |
|  | Rectal Adenocarcinoma vs. Normal | -3.998 | 0.000168 | -5.449 | Kaiser Colon |
|  | Colorectal Carcinoma vs. Normal | -5.02 | 4.63E-15 | -17.865 | Skrzypczak Colorectal 2 |
|  | Colon Carcinoma vs. Normal | -3.825 | 2.78E-09 | -15.294 | Skrzypczak Colorectal 2 |
|  | Colon Adenoma vs. Normal | -4.427 | 1.23E-06 | -8.004 | Skrzypczak Colorectal 2 |
|  | Colorectal Carcinoma vs. Normal | -5.02 | 4.63E-15 | -10.509 | Skrzypczak Colorectal |
|  | Colorectal Adenocarcinoma vs. Normal | -2.317 | 6.15E-15 | -10.407 | Skrzypczak Colorectal |
|  | Rectal Adenoma vs. Normal | -2.241 | 1.73E-06 | -8.92 | Sabates-Bellver Colon |
|  | Colon Adenoma vs. Normal | -2.153 | 4.41E-10 | -8.11 | Sabates-Bellver Colon |
|  | Rectal Adenocarcinoma vs. Normal | -6.997 | 1.66E-25 | -18.164 | TCGA Colorectal |
|  | Cecum Adenocarcinoma vs. Normal | -6.068 | 8.32E-16 | -13.026 | TCGA Colorectal |
|  | Colon Adenocarcinoma vs. Normal | -6.798 | 1.91E-23 | -20.143 | TCGA Colorectal |
|  | Rectosigmoid Adenocarcinoma vs. Normal | -7.834 | 5.44E-07 | -17.625 | TCGA Colorectal |
|  | Colon Mucinous Adenocarcinoma vs. Normal | -4.179 | 5.23E-11 | -9.159 | TCGA Colorectal |
|  | Rectal Mucinous Adenocarcinoma vs. Normal | -4.75 | 2.84E-05 | -8.286 | TCGA Colorectal |
|  | Rectal Adenocarcinoma vs. Normal | -4.449 | 1.01E-26 | -15.167 | Gaedcke Colorectal |
| KLF6 | Colorectal Adenocarcinoma vs. Normal | -2.023 | 7.65E-15 | -10.38 | Skrzypczak Colorectal |
|  | Rectal Mucinous Adenocarcinoma vs. Normal | -2.129 | 5.29E-06 | -6.977 | TCGA Colorectal |
| KLF7 | Rectal Adenocarcinoma vs. Normal | 2.598 | 5.42E-34 | 19.472 | Gaedcke Colorectal |
|  | Colon Carcinoma Epithelia vs. Normal | 4.728 | 3.23E-10 | 16.496 | Skrzypczak Colorectal 2 |
|  | Colon Carcinoma vs. Normal | 3.753 | 1.6E-10 | 17.838 | Skrzypczak Colorectal 2 |
|  | Colon Adenoma Epithelia vs. Normal | 2.348 | 1.89E-05 | 6.914 | Skrzypczak Colorectal 2 |
|  | Rectosigmoid Adenocarcinoma vs. Normal | 2.208 | 3.00-06 | 7.42 | Kaiser Colon |
|  | Rectal Adenocarcinoma vs. Normal | 2.453 | 4.1E-11 | 8.584 | TCGA Colorectal |
| KLF8 | Colon Mucinous Adenocarcinoma vs. Normal | -2.537 | 6.85E-11 | -8.457 | TCGA Colorectal |
|  | Rectal Mucinous Adenocarcinoma vs. Normal | -2.163 | 1.08E-05 | -6.395 | TCGA Colorectal |
|  | Rectosigmoid Adenocarcinoma vs. Normal | -3.664 | 0.000139 | -9.617 | TCGA Colorectal |
|  | Rectal Adenocarcinoma vs. Normal | -3.021 | 1.89E-14 | -11.54 | TCGA Colorectal |
|  | Colon Adenocarcinoma vs. Normal | -2.968 | 2.23E-13 | -12.406 | TCGA Colorectal |
|  | Colorectal Carcinoma vs. Normal | -2.448 | 1.81E-07 | -6.037 | Hong Colorectal |
|  | Colon Carcinoma Epithelia vs. Normal | -2.185 | 7.55E-05 | -5.342 | Skrzypczak Colorectal 2 |
| KLF9 | Colon Adenocarcinoma vs. Normal | -2.102 | 6.46E-05 | -4.187 | Alon Colon |
|  | Colon Adenoma vs. Normal | -3.066 | 6.55E-11 | -19.853 | Skrzypczak Colorectal 2 |
|  | Colon Adenoma Epithelia vs. Normal | -4.237 | 3.04E-09 | -13.317 | Skrzypczak Colorectal 2 |
|  | Colon Carcinoma vs. Normal | -2.667 | 1.9E-10 | -16.79 | Skrzypczak Colorectal 2 |
|  | Colon Carcinoma Epithelia vs. Normal | -3.52 | 1.32E-08 | -12.141 | Skrzypczak Colorectal 2 |
|  | Colorectal Adenocarcinoma vs. Normal | -2.584 | 2.19E-15 | -10.128 | Skrzypczak Colorectal |
|  | Colorectal Carcinoma vs. Normal | -2.504 | 7.06E-11 | -7.912 | Skrzypczak Colorectal |
|  | Colorectal Carcinoma vs. Normal | -4.631 | 3.2E-20 | -12.285 | Hong Colorectal |
|  | Colon Adenocarcinoma vs. Normal | -2.965 | 0.000132 | -4.13 | Notterman Colon |
|  | Cecum Adenocarcinoma vs. Normal | -4.13 | 3.74E-15 | -12.218 | TCGA Colorectal |
|  | Colon Mucinous Adenocarcinoma vs. Normal | -3.08 | 8.97E-12 | -9.161 | TCGA Colorectal |
|  | Rectal Adenocarcinoma vs. Normal | -5.172 | 1.26E-18 | -14.071 | TCGA Colorectal |
|  | Colon Adenocarcinoma vs. Normal | -5.093 | 1.47E-17 | -15.121 | TCGA Colorectal |
|  | Rectosigmoid Adenocarcinoma vs. Normal | -4.03 | 8.27E-05 | -9.423 | TCGA Colorectal |
|  | Rectal Adenocarcinoma vs. Normal | -2.348 | 1.24E-26 | -14.456 | Gaedcke Colorectal |
|  | Colon Adenocarcinoma vs. Normal | -2.383 | 2.91E-07 | -7.818 | Kaiser Colon |
| KLF10 | Rectal Mucinous Adenocarcinoma vs. Normal | -2.066 | 6.13E-05 | -5.265 | TCGA Colorectal |
| KLF12 | Colon Carcinoma vs. Normal | -3.536 | 1.3E-08 | -13.493 | Skrzypczak Colorectal 2 |
|  | Colon Carcinoma Epithelia vs. Normal | -5.916 | 3.81E-07 | -11.575 | Skrzypczak Colorectal 2 |
|  | Colon Adenocarcinoma vs. Normal | -2.683 | 1.56E-17 | -10.149 | TCGA Colorectal |
|  | Rectal Mucinous Adenocarcinoma vs. Normal | -2.884 | 6.95E-06 | -6.965 | TCGA Colorectal |
|  | Rectal Adenocarcinoma vs. Normal | -3.485 | 6.32E-15 | -9.432 | TCGA Colorectal |
| KLF13 | Colorectal Carcinoma vs. Normal | -2.664 | 3.09E-06 | -5.606 | Graudens Colon |
|  | Colon Adenocarcinoma vs. Normal | -2.044 | 1.33E-21 | -13.075 | TCGA Colorectal |
|  | Rectal Mucinous Adenocarcinoma vs. Normal | -2.36 | 1.98E-07 | -9.939 | TCGA Colorectal |
|  | Rectal Adenocarcinoma vs. Normal | -2.039 | 6.04E-19 | -11.703 | TCGA Colorectal |
|  | Colon Carcinoma Epithelia vs. Normal | -2.621 | 5.33E-08 | -10.628 | Skrzypczak Colorectal 2 |
|  | Colon Carcinoma vs. Normal | -2.091 | 7.94E-07 | -10.424 | Skrzypczak Colorectal 2 |
